# A novel approach to understanding Parkinsonian cognitive decline using minimum spanning trees, edge cutting, and magnetoencephalography

**DOI:** 10.1038/s41598-021-99167-2

**Published:** 2021-10-05

**Authors:** Olivier B. Simon, Isabelle Buard, Donald C. Rojas, Samantha K. Holden, Benzi M. Kluger, Debashis Ghosh

**Affiliations:** 1 Department of Biostatistics and Informatics, Colorado School of Public Health, University of Colorado Anschutz Medical Campus, Aurora, CO USA; 2 Department of Neurology, University of Colorado School of Medicine, University of Colorado Anschutz Medical Campus, Aurora, CO USA; 3 Department of Psychology, Colorado State University, Fort Collins, CO USA; 4 Department of Neurology, University of Rochester Medical Center, Rochester, NY USA

**Keywords:** Statistics, Neurological disorders, Computational neuroscience, Network topology, Computational biology and bioinformatics, Mathematics and computing

## Abstract

Graph theory-based approaches are efficient tools for detecting clustering and group-wise differences in high-dimensional data across a wide range of fields, such as gene expression analysis and neural connectivity. Here, we examine data from a cross-sectional, resting-state magnetoencephalography study of 89 Parkinson’s disease patients, and use minimum-spanning tree (MST) methods to relate severity of Parkinsonian cognitive impairment to neural connectivity changes. In particular, we implement the two-sample multivariate-runs test of Friedman and Rafsky (Ann Stat 7(4):697–717, 1979) and find it to be a powerful paradigm for distinguishing highly significant deviations from the null distribution in high-dimensional data. We also generalize this test for use with greater than two classes, and show its ability to localize significance to particular sub-classes. We observe multiple indications of altered connectivity in Parkinsonian dementia that may be of future use in diagnosis and prediction.

## Introduction

Studies of functional changes in the brain have typically focused on two distinct aspects of brain activity: regional activation, and connectivity. In the former, changes in spectral power at particular frequencies are traced to specific regions of the brain, while in the latter the focus is on inferring rates of information transfer between brain regions^1^. Modalities often employed for connectivity analysis include simple correlation, coherence, Granger causality, dynamic causal modeling, and phase transfer entropy^2^.

Graph theory, or more broadly, network science, provides a natural mathematical framework for the representation and analysis of complex networks^1,3,4^, giving it clear applicability for studies of brain connectivity. In such studies, brain regions of interest are represented as "nodes" of a graph structure, while significant information flow between the nodes is represented as connecting "edges". From this structure, useful statistical and structural principles can be derived that may give clues to pathological or functional changes^5,6^.

One approach to the study of graph connectivity is to threshold the connectivity values so that connections below a certain strength are excluded from the graph. This however introduces a subjective parameter into the analysis, which may dramatically affect results^7^. An alternative which has gained increased attention in connectivity analysis recently is the minimum spanning tree (MST), defined as the set of connections that joins all nodes of the graph into a single contiguous tree with the minimum possible total connection distance^8^. Since the MST is unique for any given arrangement of nodes and is also efficient to calculate, studies based on MST analysis are relatively free from biases due to thresholding and normalization^9–11^.

A particularly elegant application of MSTs for detecting significant differences between populations is the multivariate-runs (MVR) test of Friedman and Rafsky^8,12^, which quantifies the probability that two classes of datapoints are drawn from the same underlying distribution. Additional, better-known metrics also useful for MST-based graph analysis include edge lengths, clustering coefficients, shortest path lengths, and node degree distribution^5,6^, all of which may be combined with two-sample tests to yield highly sensitive yet tractable measures of network characteristics and changes.

Parkinson's disease (PD) is a chronic neurological condition best known for the progressive degeneration of motor neurons and concordant loss of coordination, irregularity of gait, resting tremor, and bradykinesia^13–15^; it is the second most common neurodegenerative disease in persons over age 65^3^. Neurodegeneration in PD was first observed, and has been most thoroughly characterized, in the dopaminergic neurons of the substantia nigra, a region of the midbrain that influences looped thalamo-cortical connections^16^. Degradation of these connections, in turn, is believed to play a central role both in the typical motor symptoms of PD and in executive dysfunction^14,17,18^. Most effective treatments for PD accordingly involve analogues or metabolic precursors of dopamine, particularly levodopa (l-dopa)^18^.

Although the motor symptoms of PD are perhaps the most distinctive and widely known feature of the disease, cognitive decline is also commonly observed with more advanced cases, with up to 75–83% of PD patients eventually developing dementia^19,20^. This pattern of advancing cognitive deficits, distinct from other dementia types such as Alzheimer's, is known as parkinsonian dementia (PDD). PDD manifests as "dysexecutive" difficulties in planning, abstract thinking, memory and visuo-spatial ability, with language relatively unaffected^13,17,19^, and is typically preceded by a gentler condition known as Mild Cognitive Impairment (MCI), observed in up to 38% of PD diagnoses^21,22^.

Magnetoencephalography (MEG) is a neuroimaging technique that directly observes magnetic fields produced by electric currents within the brain, using extremely sensitive superconducting magnetometers known as SQUIDs^23^. While MEG’s spatial resolution is lower than that of fMRI and it lacks fMRI’s ability to distinguish different chemical environments, MEG also has certain major advantages over fMRI, especially with regard to its temporal resolution (on the order of milliseconds) and its ability to collect electromagnetic signals directly produced by action potentials within the brain^23^. fMRI measurements, in contrast, depend fundamentally on the blood oxygen level dependent (BOLD) response, an indirect and time-lagged indicator of underlying brain activity^24^. Despite these potential advantages for brain connectivity analysis in neurodegenerative disorders, the use of MEG as an independent method for the assessment and analysis of PDD has received limited attention^25^.

In the present work, we explore the possibilities of MST-based methods, particularly the multivariate runs test, to detect group differences in medical information by performing connectivity analyses on cross-sectional resting-state magnetoencephalography (MEG) data drawn from three classes of PD patients—normal cognition (PD-NC), mild cognitive impairment (PD-MCI) and full dementia (PDD). For our connectivity analyses, we employ a simple time-lagged cross-correlation approach, based on the Pearson correlations between two MEG signals offset in time by an interval dt, to identify regions with the strongest through-time interactions in the five canonical frequency ranges. We find distinctive changes in connectivity and MST properties as a function of disease severity, as well as changes in variability in connectivity among patients. Further, by applying a modified version of the MVR test that generalizes it to operate on more than two classes, we find strong confirmation that our three cognitive groups arise from highly distinct distributions. This suggests the possible application of our multi-class MVR approach to many other contexts involving hypothesis-testing in high-dimensional medical data.

## Methods

### Participants

Eighty-nine subjects with Parkinson’s disease were recruited from the University of Colorado Hospital Movement Disorders clinic as well as by fliers posted on campus. All gave informed consent to participate, and the study was approved by the Colorado Multiple Institution Review Board. All methods were performed in accordance with the relevant guidelines and regulations. Inclusion depended on a diagnosis of probable PD in accord with UK Brain Bank Criteria^26^. PD subjects with features suggestive of other causes of parkinsonism, cerebrovascular disease, a history of major head trauma, or a history of deep brain stimulation or ablation surgery, were excluded. All subjects were examined while in the dopaminergic “ON” state. Table 1 describes participants’ demographics and baseline clinical features.

**Table 1:** Participant demographics and clinical features.

Demographics	PD-NC	PD-MCI	PDD	F/*t*/χ^2^-value	p-value
Sex (male)	25/37	26/35	12/17	5.115	0.059
Age	69.74 ± 6.95	66.71 ± 6.86	75.78 ± 8.59	8.029	**0.001**
Education (years)	16.48 ± 4.05	15.76 ± 4.21	16.81 ± 3.03	0.523	0.568
Handedness (right)	31/37	28/35	15/17	0.728	0.402
Levodopa equivalence	374.17 ± 385.97	586.37 ± 421.17	753.61 ± 481.08	4.230	**0.018**
UPDRS total	39.04 ± 6.94	43.60 ± 12.24	170.22 ± 17.69	740.45	**0.00001**
Hoehn and Yahr	2.16 ± .27	2.23 ± .56	2.75 ± .72	3.294	0.059

Values are given as fractions, or means ± SD. The levodopa equivalence standard deviation is high in our cohort, indicating that participants used a broad range of dosages. “UPDRS” = Unified Parkinson’s Disease Rating Scale.

The p-values in bold indicate those differences between the populations that are statistically significant at a 0.05 level of significance.

### Cognitive assessment and classification

The three PD groups were defined according to their level of cognitive abilities: Normal Cognition (PD-NC; n = 35), mild cognitive impairment (PD-MCI; n = 37), and Parkinson’s disease dementia (PDD; n = 17). PD-MCI diagnosis was modeled on Movement Disorders Society (MDS) Task Force diagnostic criteria, requiring scores greater than one standard deviation below norms on at least two neuropsychological tests. Comprehensive neuropsychological test batteries used included the Montreal Cognitive Assessment^27^, the Boston Naming Test^28^, the Dementia Rating Scale-2^29^, Trails Making Test^30^, the Brief Test of Attention^31^, the California Verbal Learning Test, Second Edition ^32^, the Symbol Digit Modality Test (SDMT) Oral^33^, the Delis-Kaplan Executive Function System (DKEFS) Verbal Fluency/Letter Fluency task^34^ and the Judgment of Line Orientation task (JLO)^35^. These tests were chosen based on previous work operationalizing the PD-MCI criteria^36^.

Classification of PD-NC, PD-MCI, or PDD was determined by consensus conference, attended by a neurologist and a neuropsychologist with experience in PD cognition. Prior to consensus conference meetings, raw scores were transformed to z-scores based on normative data for each of the individual neuropsychological tests (see next paragraph for a list of the tests), drawn from either testing manuals^32,37–43^ or additional normative studies^44–47^. MDS Task Force Level II diagnostic guidelines for PDD^48^ and PD-MCI^49^ were applied, requiring impairment on two tests in one cognitive domain (PD-MCI) or one test in each of two cognitive domains (PD-MCI, PDD) for classification as cognitively impaired. Of note, we diverged from the MDS recommendations in that we did not have two neuropsychological tests for all domains tested (e.g. visuospatial abilities). Impairment was defined as performance of ≥ 2 standard deviations below age- and education-matched norms^36^. PD-MCI was differentiated from PDD based on the results of the neurologist’s functional interview: if significant functional impairment related to cognitive symptoms was present based on clinical impression, a participant was classified as PDD.

Cognitive data were found to violate the missing completely at random (MCAR) assumption (Little’s MCAR Test, p = 0.007). To address this, data were imputed to create five multiple imputations; this imputed data did not differ significantly from the original data (all ps > 0.05). Cognitive composite scores were created for data reduction, using PCA for each set of imputed data separately. Composites were created from two variables per domain as follows: Global cognition, MoCA and DRS-2 total scores; Attention, TMT-A and BTA; language: BNT and verbal fluency; learning and memory, CVLT trials 1–5 total score and CVLT long delay free recall; executive function, TMT-B and SDMT oral. As the JLO Test was the only test of visuospatial abilities, a composite score was not created.

### MEG data acquisition and preprocessing

Resting-state data was acquired continuously at 678.17 Hz using a Magnes 3600 whole-head MEG device with a 248-sensor array (4D Neuroimaging, San Diego, CA) in a magnetically shielded room (ETS-Lindgren, Cedar Park, TX, USA). Acquisition bandwidth was 0.1–200 Hz. Location and orientation of the MEG coils relative to each subject’s head were determined by digitizing fiducial reference points on the head with a Polhemus 3SPACE magnetic digitizer. Left and right preauricular points and the nasion were digitized as reference points and the shape of each participant’s head was digitized for use in constructing a volume conductor model for source localization. Next, data was acquired for a minimum of 4 min, with subjects in supine position. Subjects were instructed to keep their eyes closed for the first half of the acquisition period, and eyes open for the second half, as announced by a brief sound (2 kHz sine wave). Eyes-closed and eyes-open data were combined, while the period in which the sine wave was played was excluded.

Time-series MEG spectral power and source estimates of brain activity were processed with a custom pipeline in MATLAB 2016b (MathWorks, Inc., Natick, MA, USA) using the FieldTrip toolbox for MEG analysis^50^). Raw MEG data underwent bandpass filtering between 0.5 and 55 Hz using a 4th order, phase-invariant Butterworth characteristic filter. MEG channels showing excessive (> 2 SD) variance in signal intensity and spectral power relative to neighboring sensors were dropped; these, in turn, were visualized using heat-maps according to PD group, to check for class biases with regard to channel removal (Supplementary Figure S1). Additionally, MEG channels jump artifacts were identified and excluded, using the FieldTrip ft_artifact_jump.m function. Eye blink/movement and electrocardiogram artifacts were identified and removed by Independent Components Analysis (ICA) using the ‘runica’ algorithm^51^, with the number of ICA components limited to 50 using principal components analysis. The ICA-cleaned data was next subjected to discrete Fourier analysis, yielding a power spectrum over 99 frequencies over the range 0–50 Hz, encompassing the five main brainwave frequency bands (delta, 0.5–3.5 Hz; theta, 4–7 Hz; alpha 8–12 Hz; beta, 13–30 Hz; and gamma, 31–55 Hz). The data were simultaneously formed into 2-s contiguous trials to allow resolution down to 0.5 Hz, with tapered 0.5 s overlaps to reduce data loss from spectral leakage, giving an average interval between trials of 1.5 s. Trials with amplitudes above ± 3 pT were excluded from further analysis.

### MEG data analyses

3D source reconstruction was carried out with a standardized MRI headmodel (standard_singleshell.mat from FieldTrip) in Montreal Neurological Institute (MNI) space; this model was used for all patients. Using the Matlab SPM 12 toolbox function spm_eeg_inv_rigidreg.m (SPM12; http://www.fil.ion.ucl.ac.uk/spm/), the MNI head model and an 8-mm cubic voxel, MNI space source model (standard_sourcemodel3d8mm.mat) were co-registered to the individual subjects using the MEG fiducials for each subject and corresponding MRI fiducials. Leadfields were then computed from the source model, and source analysis was conducted with the FieldTrip ft_sourceanalysis function.

To obtain the MEG time-series used for the connectivity analysis, minimum-norm estimation ('mne') was used for the source modeling^23^. In order to achieve a resolution commensurate with subsequent brain parcellation, interpolation was performed on the source model for each trial, increasing its dimensions from 20 × 22 × 25 to 91 × 91 × 109. Voxel power was normalized by dividing the power of the trials with above-median power by those with below-median power (hereafter referred to as “ratio” power).

Volumetric models of brain activity were analyzed with respect to functionally organized brain "parcels" rather than individual voxels. Parcellation of the source models was carried out with the Anatomical Automatic Labeling (AAL) parcellation^52^, included in FieldTrip/MATLAB. This parcellation was chosen because it is readily available in FieldTrip and has been successfully used in prior PD studies^20^. In addition, its parcel sizes are large enough to be resolvable at spatial resolutions typical of MEG reconstruction. Analysis of the resulting parcellated 3D activation data was also carried out in MATLAB. For each trial, each parcel’s ‘power’ was calculated as the average of the ratio powers of its constituent voxels for the brainwave frequency band of interest.

### Minimum spanning tree (MST) analysis

Once average power had been calculated for each parcel over successive trial intervals, Pearson correlations were computed between the parcels with a specified time offset between them, yielding a 116 × 116 matrix of time-lagged cross-correlations^53,54^. Parcel correlations were calculated for time offsets of dt = 0, 1, and 2 trials (0, 1.5, and 3 s). To visualize the inter-parcel connectivity patterns, MSTs were constructed among the correlation observations between the parcels in the following way. First, for each element of the time-lagged correlations matrix, absolute values were subtracted from 1, yielding a "distance": inter-parcel correlations of either 1 or − 1 thereby correspond to "zero distance" between parcels, while 0 correlation yields the maximum distance. This distance matrix was then symmetrized by choosing the shortest distance value between cross-diagonal pairs (i.e., the dominant direction of information flow between each parcel pair). MSTs were then generated from this distance matrix using MATLAB’s minspantree function.

MSTs were computed individually for each subject, as well as from correlations averaged over each of the three PD classes. In this way, differences between the three cognitive classes could be assessed by aggregating characteristics from individual subject MSTs within classes. MST characteristics sampled included (1) pooled edge distances; (2) node degree distribution; (3) leaf count (number of nodes with degree = 1); (4) Average shortest path length, and (5) clustering coefficients, as defined in^6^. The Kolmogorov–Smirnov (KS) two-sample test, a simple, omnibus and widely-used non-parametric test for identifying significant differences between distributions, was used to compare distributions over the three possible pairings of disease classes and evaluate significance.

Differences in the structure of inter-parcel correlations were also assessed using the two-sample Multivariate Runs (MVR) Test^8,12^. In MVR, data-points from two different populations are pooled, and an MST is constructed connecting them. Hybrid edges of the MST (i.e., those joining dissimilar classes) are removed, and the test statistic, R, is the resulting number of disjoint trees plus 1.

From^8^ it is known that in the null case for a pair of MSTs with N_a_ and N_b_ nodes respectively (and N = N_a_ + N_b_), R is normally distributed with mean equal to

$${\text{E}}\left[ {\text{R}} \right] = {\text{ 2N}}_{{\text{a}}} *{\text{N}}_{{\text{b}}} /{\text{N }} + { 1}{\text{.}}$$

The variance of R, dependent on the particular structure of the given MST, is equal to

$${\text{var}}\left[ {{\text{R}}|{\text{C}}} \right] \, = {\text{ 2N}}_{{\text{a}}} *{\text{N}}_{{\text{b}}} /{\text{N}}/\left( {{\text{N}} - {1}} \right)*\left\{ {\left( {{\text{2N}}_{{\text{a}}} *{\text{N}}_{{\text{b}}} - {\text{N}}} \right)/{\text{N}} + \left( {{\text{C}} - {\text{N}} + {2}} \right)/\left( {{\text{N}} - {2}} \right)/\left( {{\text{N}} - {3}} \right)*\left[ {{\text{N}}\left( {{\text{N}} - {1}} \right) - {\text{4N}}_{{\text{a}}} *{\text{N}}_{{\text{b}}} + {2}} \right]} \right\}.$$

Here the quantity C refers to the number of pairs of edges within the pooled MST that share a common node. The extent to which this statistic diverges from the null expectation then allows significance to be easily calculated. MVR was carried out for each combination of time offset (dt) and frequency band, for each of the three possible pairs of disease classes (N–M, M–P, N–P) were pooled, and a 2-class MST was generated over all correlation-vector endpoints in the pair of classes.

As our data involved three rather than two disease classes, we developed a 3-sample extension of the MVR method, using a pooled MST drawn over all three classes. Relationships between the classes in this pooled MST are stored in a “class-adjacency matrix”, where the entry a_ij_ contains the number of edges in the MST that link nodes from class i and j. In this scheme, the number of same-class edges is on the diagonal and the number of hybrid edges on the off-diagonal positions.

The expected variance in the total number of trees, as well as each individual edge type a_ij_ in the pooled 3-class MST after cutting dissimilar edges, is estimated by randomly permuting the node class labels of the pooled 3-class MST, and storing the number and type of edges in the resulting permuted MST in a class-adjacency matrix. This node-label permutation process is repeated, each time creating a new set of class-adjacency values, until the variance under the null for each type of edge was estimated to desired precision (for these experiments, we used 50,000 iterations). Subtree counts from MVR for each iteration are then easily derived from the diagonal entries of the class-adjacency matrix and the number of observations in each class, because of the fact that

$${\text{S}}_{{\text{k}}} = {\text{ N}}_{{\text{k}}} {-}{\text{ E}}_{{{\text{kk}}}}$$

where S_k_ is the number of subtrees of class k after cutting all MST hybrid edges, N_k_ is the number of nodes of class k in the MST, and E_kk_ is the number of same-class edges of class k. (In other words, the number of trees resulting from MVR is the total number of observations minus the trace of the class-adjacency matrix.) Simulated standard deviations for all combinations of dt and brainwave frequency band were remarkably consistent at ~ 8.75, indicating that the differences in MST structure had a minimal effect on the variance of the 3-way MVR cut.

We also constructed and visually inspected the cross-connectivity matrices for each disease class with respect to both mean correlation and correlation variability. To take into account the possibility that age or levodopa dosage could influence our results, a median split was performed to divide the patients for each cognitive class into “above-median” and “below-median” subgroups with respect to these two variables; our connectivity analysis was then repeated on each subgroup (Supplementary Figure S2 and Supplementary Figure S3).

Relevant MATLAB scripts include OS_MEGmakeCircularCorrMSTs02.m, which makes the time-lagged cross-correlation matrix, draws MSTs, calculates MST features, and carries out MVR; and OS_MultivariateRuns_varianceByPermuting.m, which carries out the 3-class MST class-adjacency variance estimation. All scripts are available online at https://github.com/Ghoshlab/OsimonScripts/tree/main/ConnectivityPaper.

## Results

We first used time-lagged cross-correlations^53,54^ to assess brain-parcel connectivity in terms of (1) pooled edge distances; (2) node degree distribution; (3) leaf count (number of nodes with degree = 1); (4) Average shortest path length, and (5) clustering coefficients. We then carried out the MVR test on the overall MST and also investigated class-adjacency information in the MST from all three cognitive classes pooled.

### General properties of MSTs drawn from parcel correlation matrices

**Figure 1:**
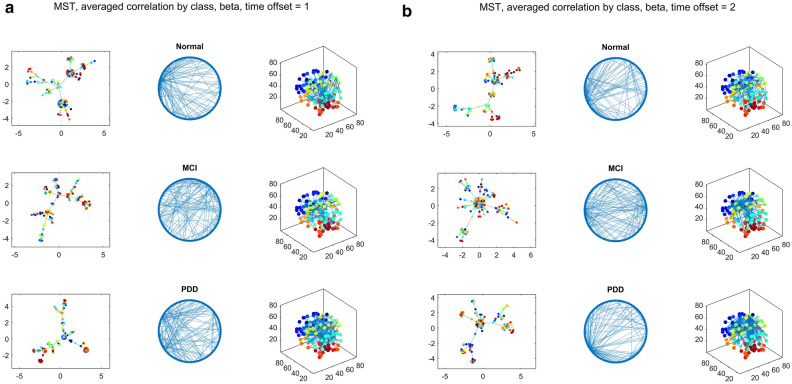
Examples of class-averaged parcel-correlation MSTs for beta-band frequencies and time offsets of dt = 1 (**a**) or dt = 2 trials (**b**). The left columns show the MSTs in a force-directed 2D layout, with nodes colored by parcel identity; middle columns show MSTs in a standard circular format which allows visual comparison; right columns show the locations of MST connectivity with respect to parcels’ positions in the brain. Substantial differences in overall connectivity are visible between the three disease classes, “normal cognition”, “MCI”, and “PDD”. PDD tends to show a more disorganized pattern in this band, with less prominent high-degree “hub” regions.

Properties of MSTs for each patient were aggregated by cognitive class. As might be expected, there is a general tendency towards weaker correlations as the time offset (dt) is increased. For simultaneous correlations (dt = 0), parcel correlations tend to be high (0.5–1) and exhibit a standard connectivity pattern with little variation, with a basal-to-frontal general orientation likely dominated by the physical adjacency of the parcels. Therefore, for the dt = 0 case, each patient’s correlation structure, the average difference between adjacent and non-adjacent parcel correlations was calculated and subtracted from the adjacent correlations. For intermediate time offsets (dt = 1), correlations are smaller (0.1–0.5), but show more differences between frequency bands (Fig. 1a). For larger time offsets (dt = 2), the parcel correlations become much smaller, and the MSTs themselves show very little apparent common structure (Fig. 1b). With time-lagged correlations (dt > 0), MSTs instead begin to exhibit more prominent "hubs" (nodes of very high degree). In the theta band for example, with a time offset of dt = 2 trials, the PD-MCI group shows a single hyper-connected node at the right angular gyrus (Angular_R), whereas the PD-NC and PDD groups show a more mixed distribution of moderately connected nodes.

The distribution of MST edge lengths showed highly significant changes (often p < 10^–8^) with cognitive class in nearly all combinations of frequency band and time-offset (Supplementary Table S1); moreover, significance tended to increase with larger time offset. Node degree distributions, on the other hand, generally did not show significant differences, with the exception of the theta band where dt = 1, 2. After applying a Bonferroni correction for the number of comparisons (3 class pairings × 3 time offsets × 5 bands = 45), only the node degree differences in theta, dt = 1 between NC and MCI and between NC and PDD remained significant at a 0.05 overall significance level (p < 8.4 × 10^–4^ and p < 6.2 × 10^–3^, respectively).

**Figure 2:**
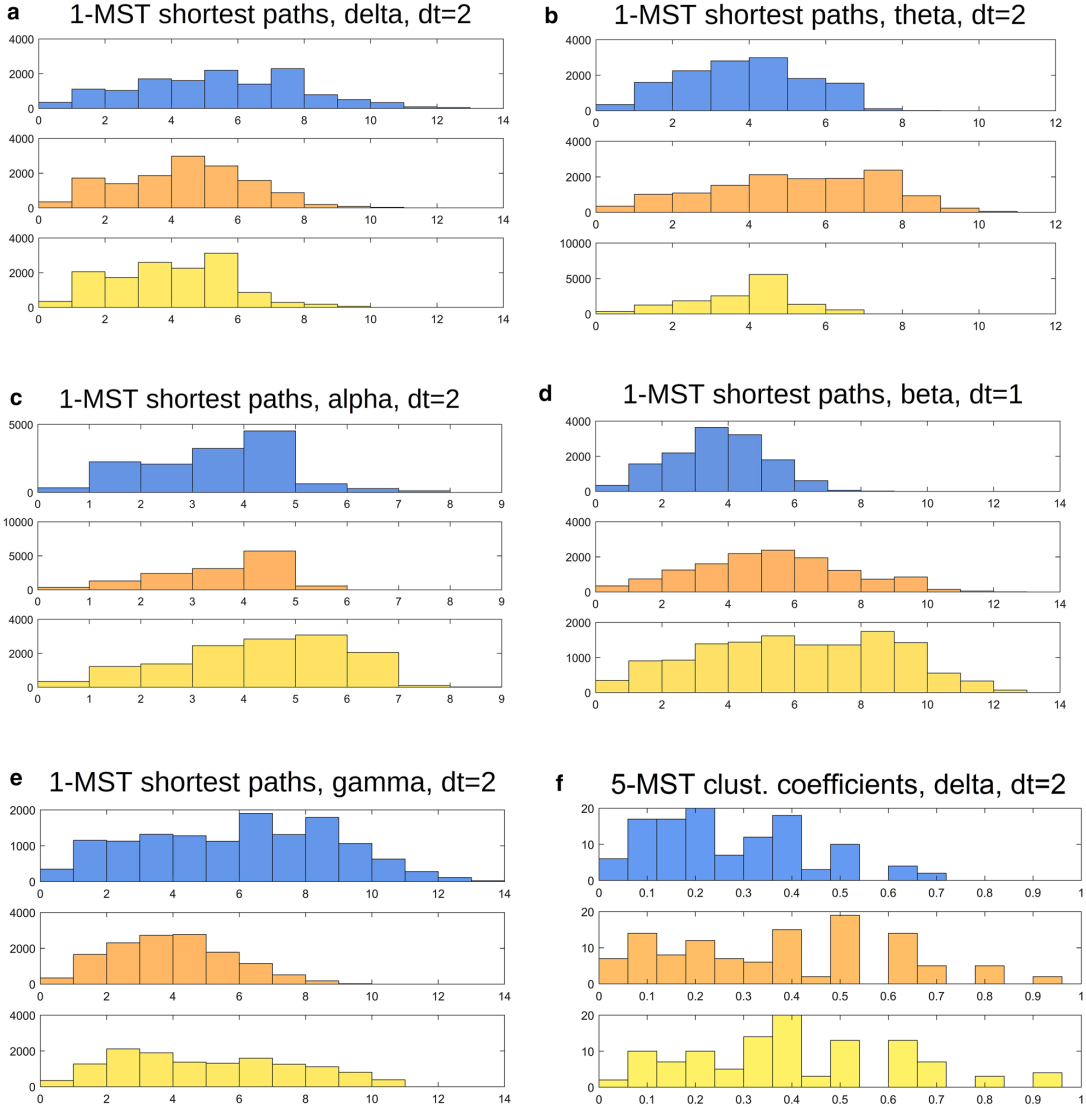

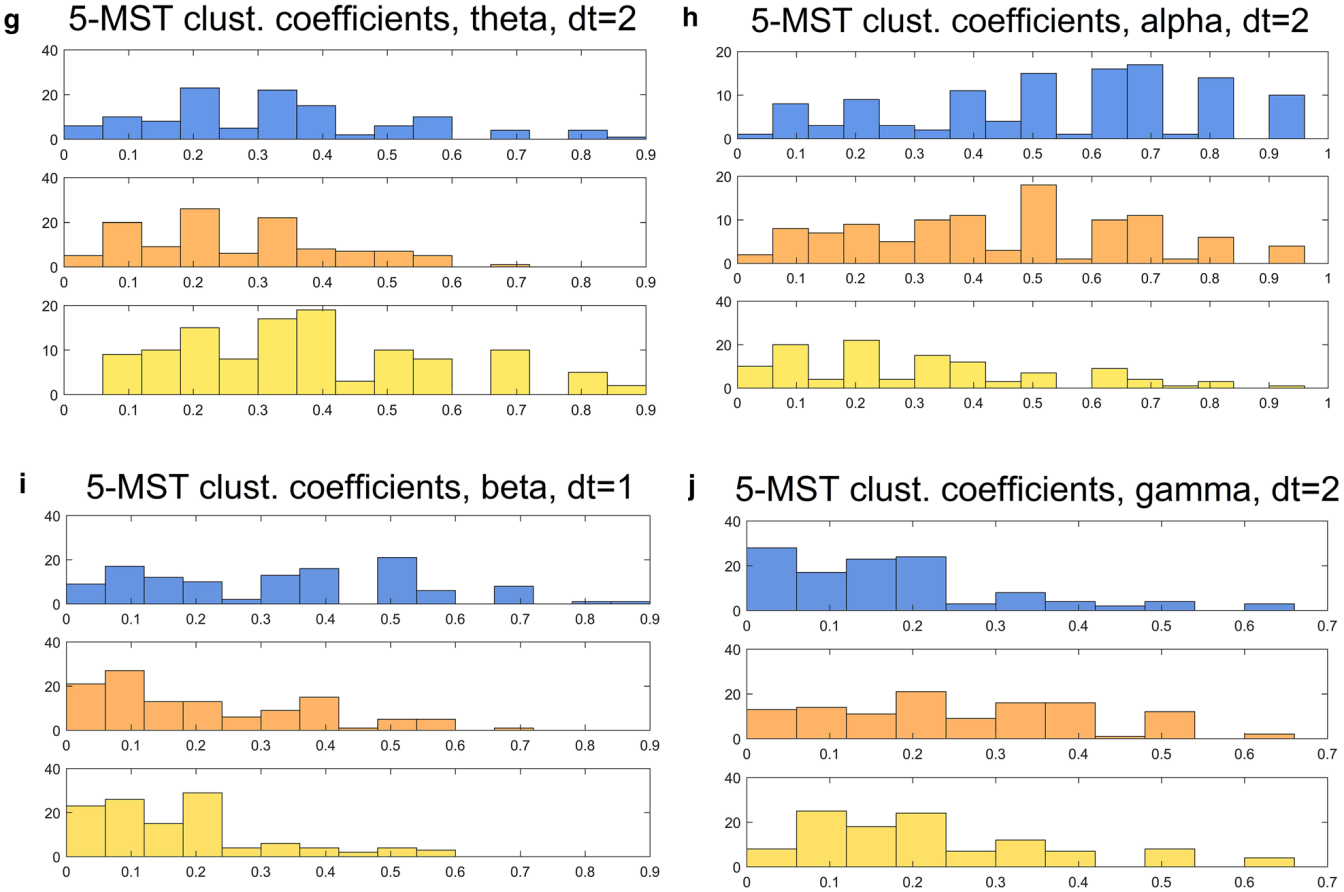
Histograms of MST shortest-path-length and clustering coefficient histograms (left and right columns, respectively) for the five combinations of time-offset and frequency band that show particularly strong changes in distribution with disease class (delta, dt = 2; theta, dt = 2; alpha, dt = 2; beta, dt = 1; gamma, dt = 2). Beta (and to a lesser extent alpha) shows a steady increase in the overall path-length with disease severity, along with increased variance. Delta, by contrast, shows decreasing path-length and variance with severity. Trends for clustering coefficients tend to be roughly inverse to those in path-lengths.

Histograms of MST shortest path-lengths between parcels, aggregated by class, show some dramatic differences between cognitive classes. The lower-frequency bands, (delta and theta) show differing patterns, with delta gradually increasing with cognitive decline (Fig. 2a) and theta gradually decreasing in MCI (Fig. 2b). For the alpha band, there is a suggestion of an increase in shortest path-lengths in PDD relative to MCI and NC (Fig. 2c). In the beta band, the distribution of shortest path-lengths shows, with worsening cognitive impairment, both an overall increase in shortest path lengths and an increase in variance of these distances (Fig. 2d). The shortest path-lengths for the gamma band, by contrast, show decreasing path-lengths and a decrease in variance, with partial recovery in PDD (Fig. 2e). In several cases, the KS tests yielded p-values so low as to be effectively zero (Supplementary Table S1). The distribution of clustering coefficients, meanwhile, tracks roughly inversely to the shortest path-lengths (Fig. 2f–j).

In general, there is no clear systematic change in the MSTs' overall shape across bands, dementia classes, or time-lags, but the significant changes in the distributions of MST edge lengths may give clues to dementia-related changes in functional connectivity, particularly in the bands.

### MVR for correlation MSTs

**Figure 3:**
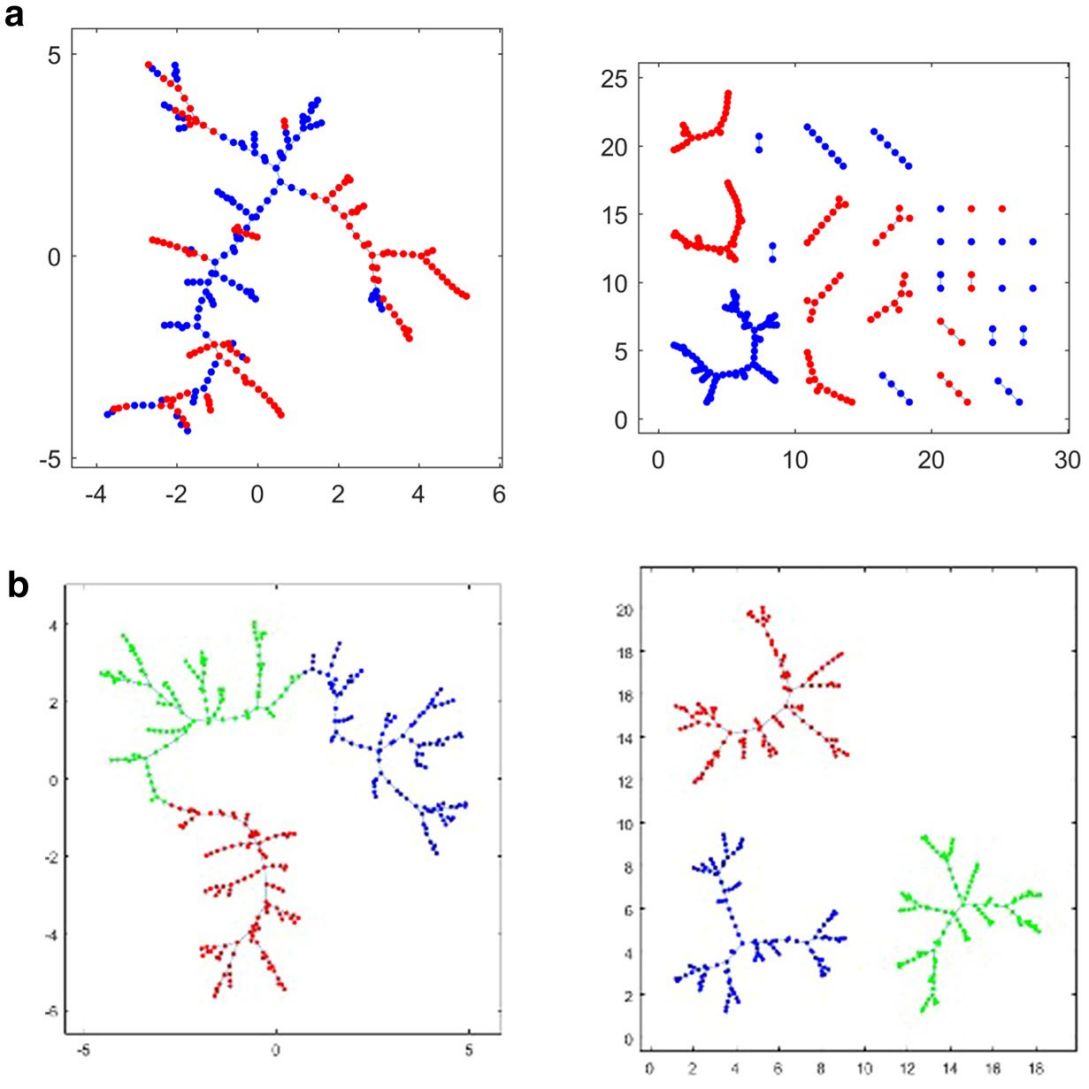
Illustrations of the multivariate-runs (MVR) MST-cutting procedure. The nodes for different disease classes are pooled, yielding and MST composed of a mixture of class characteristics (left panels). The MST edges between dissimilar node-classes are then removed, yielding disjoint subtrees (right panels). The test statistic is the number of trees produced; in general the fewer the subtrees, the less probable is the null hypothesis. (**a**) Presents an example with the classic 2-class test; (**b**) Shows results from pooling all three disease classes.

**Figure 4:**
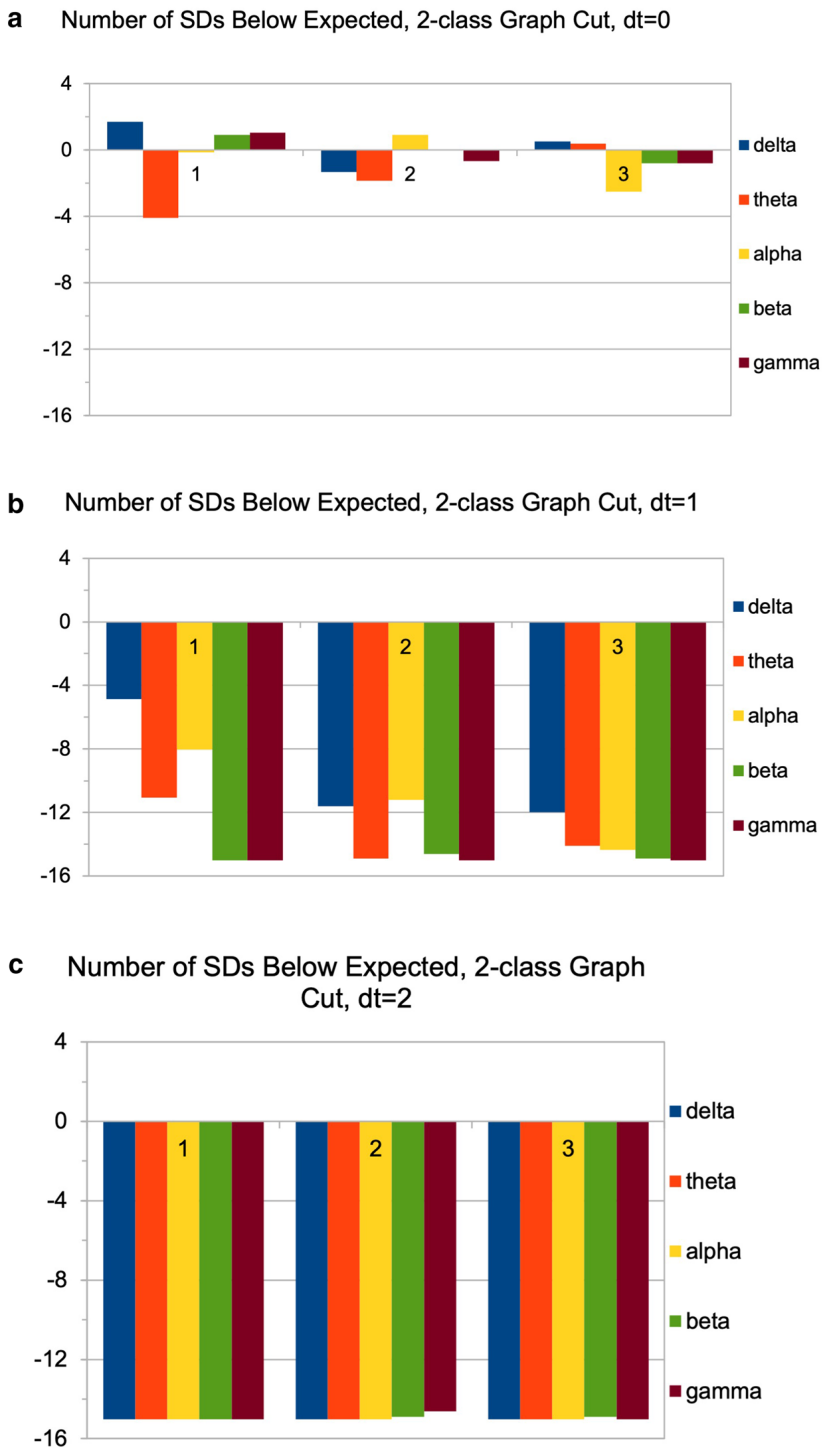
MVR 2-class edge-cut results, presented in terms of number of standard deviations from the null expected value. Graphs represent deviations for (**a**) dt = 0; (**b**) dt = 1; and (**c**) dt = 2. In general, all bands and all class pairings show increasingly significant deviations with increasing time-delay. Bars are grouped as follows: ‘1’ = ‘NC-MCI’; ‘2’ = ‘MCI-PDD’; and ‘3’ = ‘NC-PDD’.

For all three values of dt and all five frequency bands, MVR was carried out using the correlation data as position vectors (Fig. 3; additional examples shown in Supplementary Table S2). 2-class test results showed highly significant differences in the MST between all three cognitive classes, indicating that their connectivity patterns derive from very significantly different distributions (often > 10 SD or more) from the expected value (Fig. 4). 3-class MST cuts showed similarly vast deviations from the null hypothesis, with far fewer trees after edge-cutting than reference distributions would predict (data not shown). Deviations were generally largest with NC-PDD pooled MSTs, and notably increased with time offset, so that for dt = 2 most bands and class combinations showed essentially the largest possible deviation from the null. Curiously, few strong differences were found between frequency bands in this respect: all five showed very large deviations from the expected value when the cognitive classes were compared.

To test that these very high significances were indeed representative of differences between the cognitive groups, MVR was also carried out on simulated distance matrices containing only random Gaussian values, as well on actual distance matrices with trials randomly assigned to disjoint "cognitive groups" of 3000 trials. In both cases, MVR yielded fragment-counts far higher than those based on actual correlation data or dementia class groupings, and indistinguishable (i.e., within 1 SD) from the expected null value. This confirms that MVR can be used to detect substantial cognitive class-based differences within the parcel correlation structure.

One concern in these analyses is the possible confounding effects of age and levodopa treatment. By dividing the patient classes according to age and levodopa intake, we found that the MST-based measures were considerably more robust to such potential confounders than the purely correlation-based maps (Supplementary Figure S2 and Supplementary Figure S3).

### MST edge-adjacency effects

**Figure 5:**
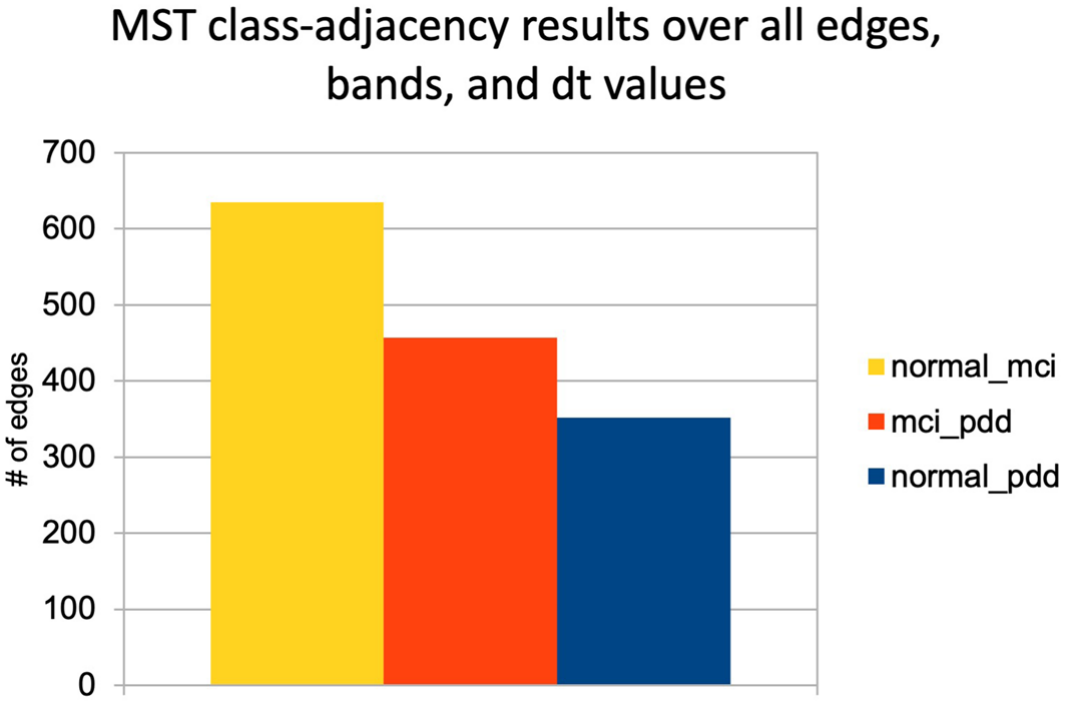
Class adjacency results for 3-class MSTs, aggregated for all bands and time-offset values. Overall, nodes from PD-NC and PD-MCI parcels are the likeliest to share edges in the combined MST, suggesting that these two groups are closest in overall connectivity structure. The pairing of PD-NC and PDD, representing the most drastic difference in cognitive function, also shows the lowest adjacency.

In considering the properties of the pooled MST generated by the MVR test, we wondered whether the distribution of the MST edge-types might carry useful information regarding the relative proximity or similarity of the 3 cognitive classes. Therefore, we binned the MST edges according to the class identity of both nodes. Aggregated over all conditions, we found nodes from the PD-NC and MCI classes were most likely to share edges in the 3-class MST, while PD-NC and PDD were much less likely; the number of shared edges between PD-MCI and PDD were intermediate (Fig. 5). Viewed individually, the alpha, beta and gamma bands concurred with this overall pattern, while theta showed the opposite trend with PD-NC and PDD most likely to share edges; delta was intermediate, with NC-MCI class adjacency the highest but MCI-PDD adjacency somewhat lower than NC-PDD (Supplementary Table S3).

### Correlation matrix visualizations

**Figure 6:**
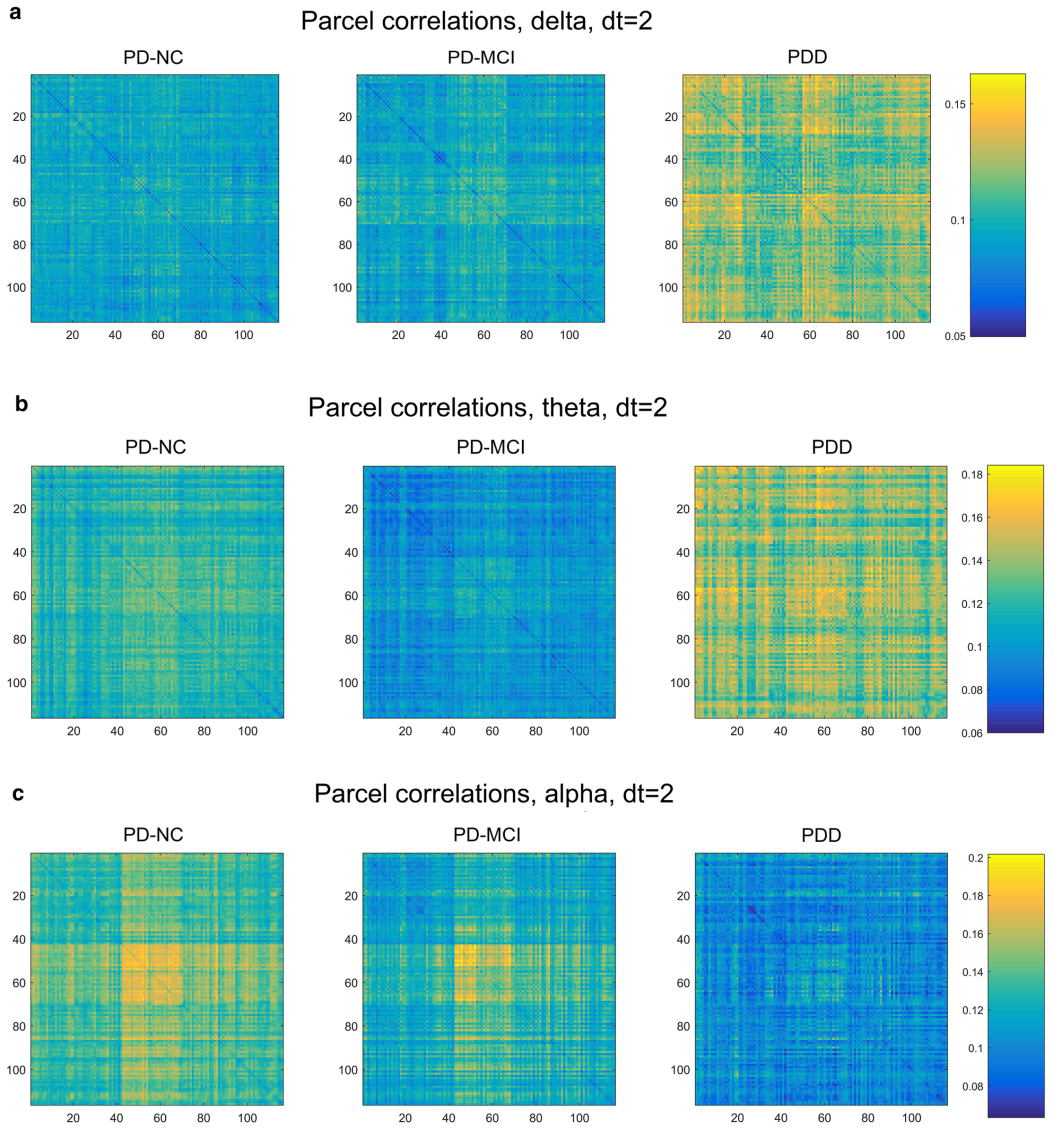

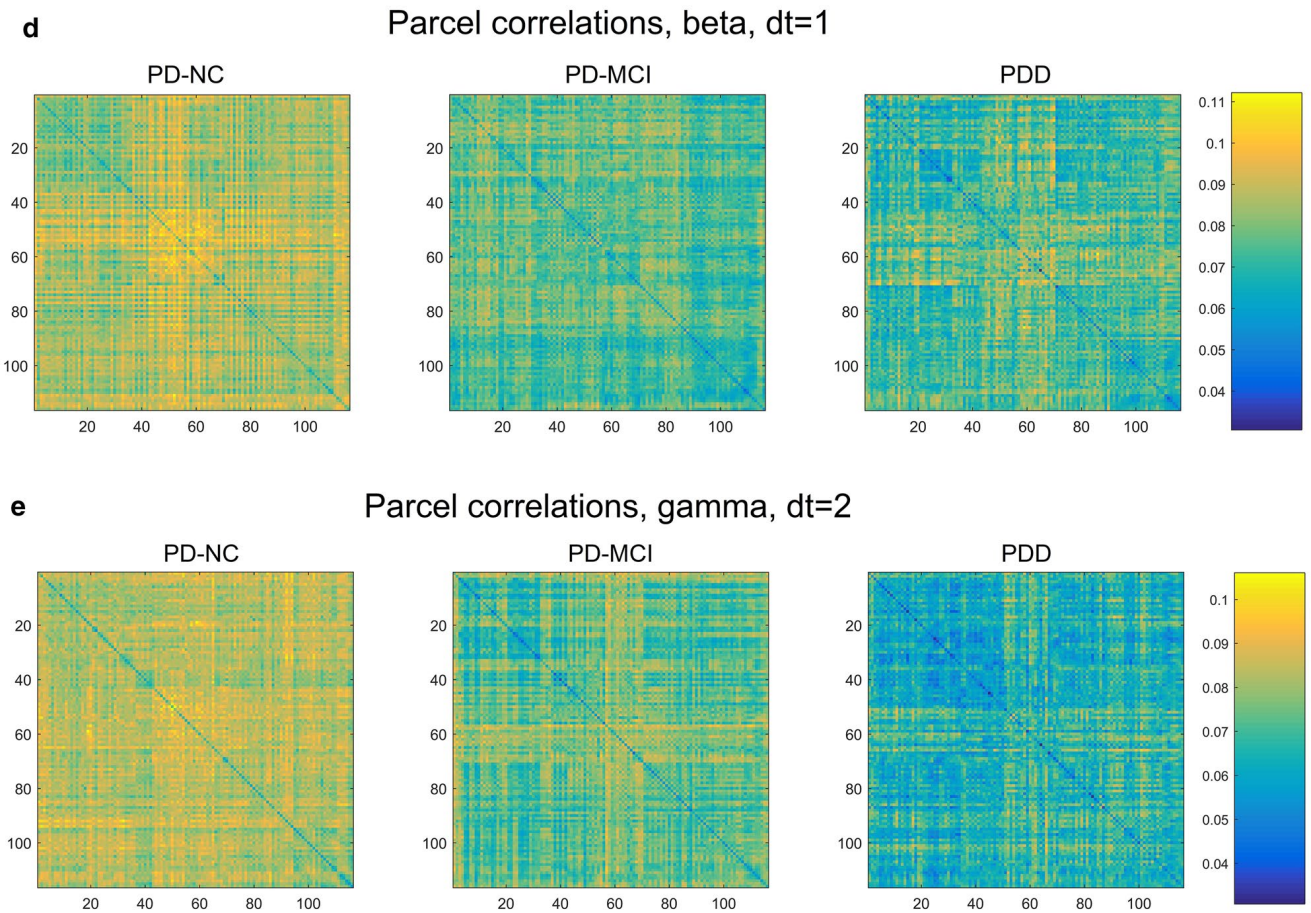
AAL parcel-correlation maps showing most substantial correlation differences among the five frequency bands [(**a**) delta, (**b**) theta, (**c**) alpha, (**d**) beta, (**e**) gamma]. Vertical axis represents the earlier state of the parcel, while horizontal axis represents the later.

Maps displaying the correlation structure among the 116 AAL parcels, averaged over each cognitive class, are shown in Fig. 6. These allow a more intuitive, visual impression of which subsets of parcels show significant correlation, while including correlations that were necessarily excluded in the creation of the MST. Note that the vertical axis indicates parcels in the "previous state", while the horizontal indicates these parcels in the "subsequent state" (or, in the Granger sense, "cause" and "effect")^2^.

For the delta and theta bands, we observe a strong increase in overall correlation with worsening cognitive decline (Fig. 6a,b), suggesting that the increase in low-frequency power also is marked by increased connectivity for such power essentially brain-wide. By contrast, this trend is reversed in the alpha band, particularly dt = 2 (Fig. 6c), so that cognitive decline appears to involve decreased connectivity. Here, the PD-NC group shows strongly heightened correlations in both hemispheres between the calcarine sulcus (primary visual cortex), cuneus, lingual gyrus, postcentral gyrus, supramarginal gyrus, angular gyrus, and precuneus, which greatly decrease with cognitive decline. This correlation pattern is also detectable, though much less distinctly, in the beta band, though on the whole the two higher-frequency bands show a much more global reversal of the low-frequency correlation trend, with the beta and gamma showing a strong decline in correlation with increasing dementia severity. With the exception of this group of alpha-generating regions, no distinct groups of parcels acting together as "sub-networks" or "resting state networks" were apparent.

**Figure 7:**
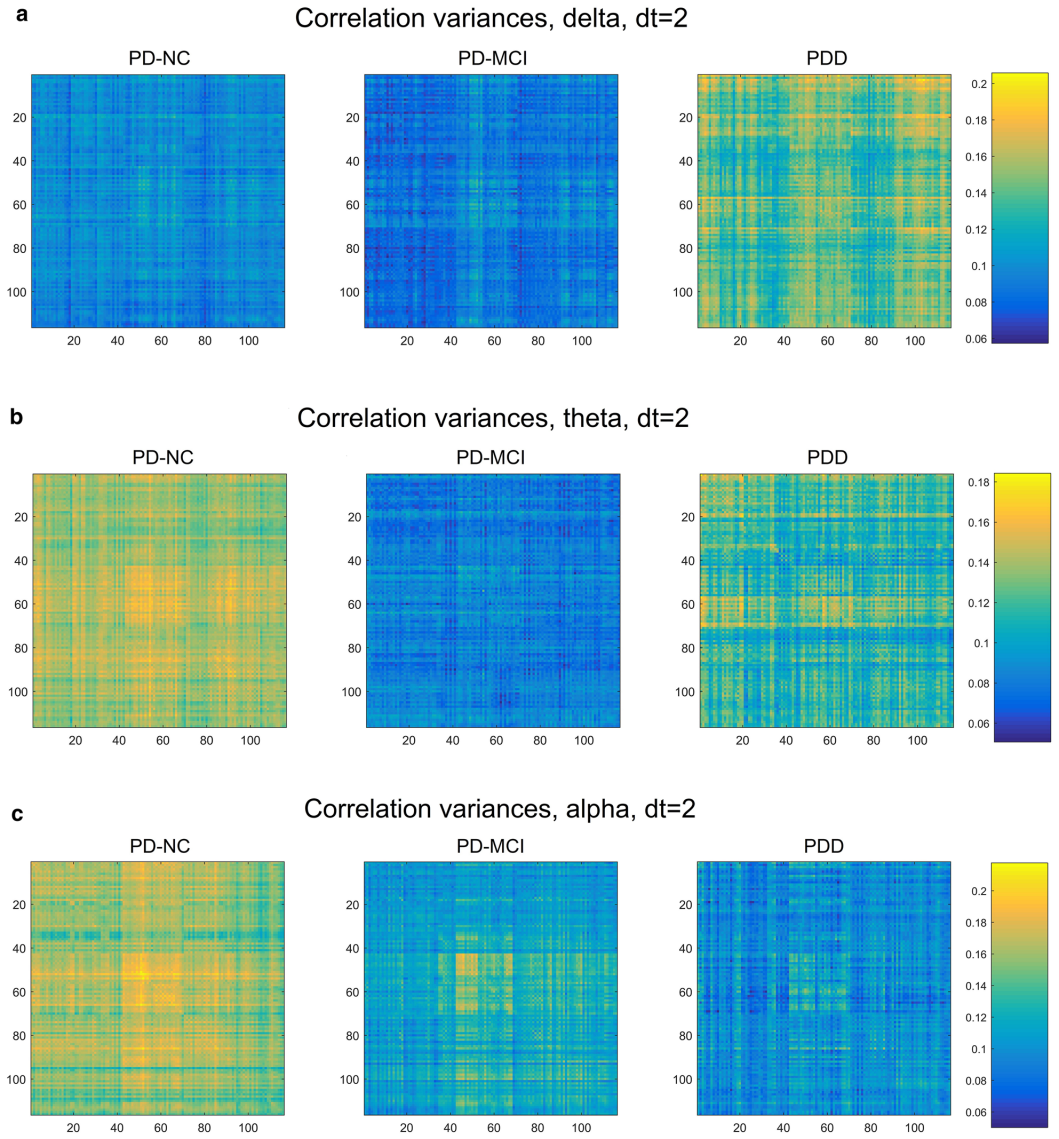

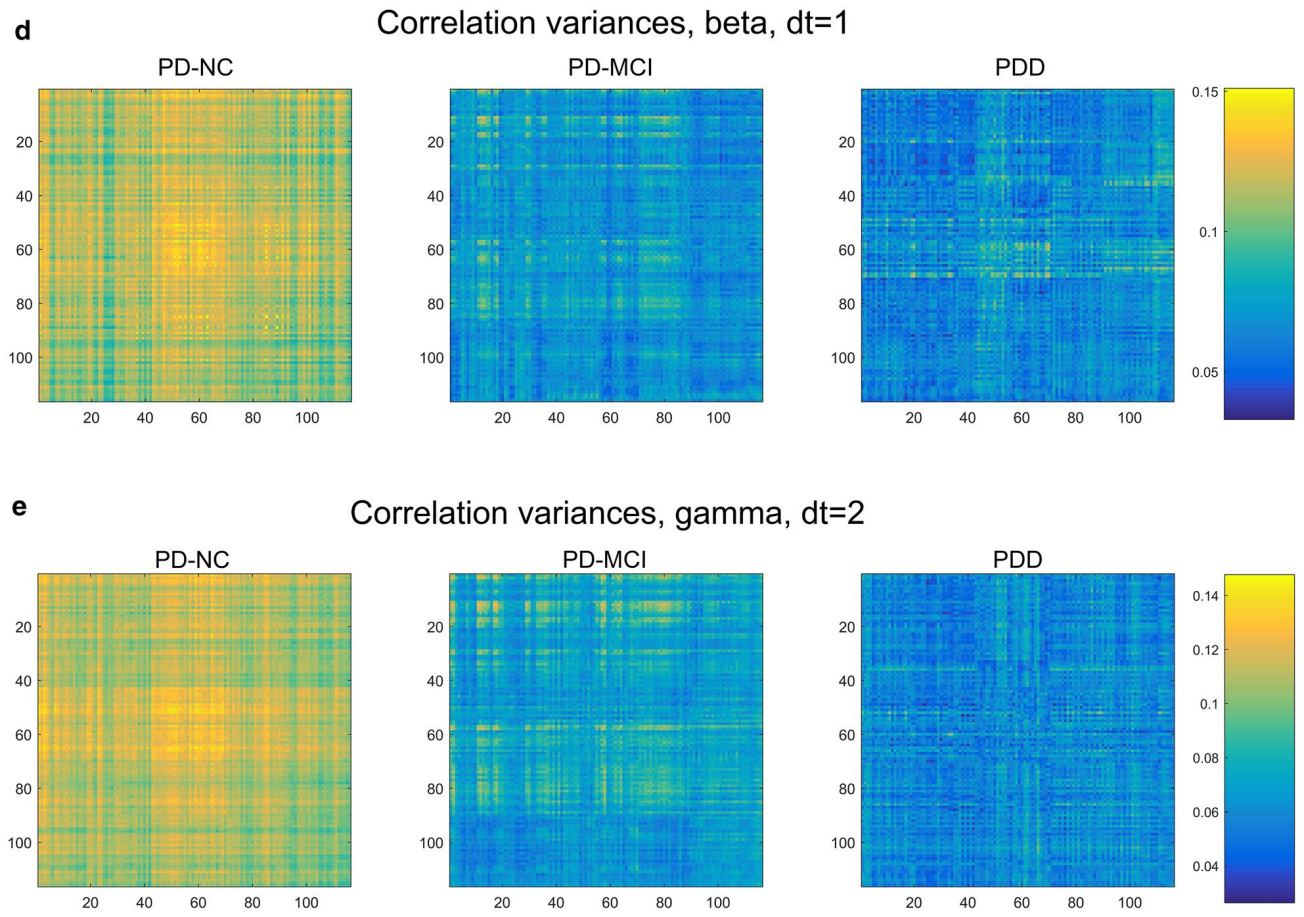
Correlation variance maps. For all brain-wave frequency bands except delta, there is a marked, mostly global drop in correlation variability from PD-NC to PD-MCI groups [(**a**) delta, (**b**) theta, (**c**) alpha, (**d**) beta, (**e**) gamma]. The two lower-frequency bands show a partial recovery in variability in the PDD cases.

In addition to correlation itself, maps of inter-subject variance in correlation were also generated (Fig. 7). Interestingly, we found strong indications in all bands that connectivity becomes considerably less variable between patients with dementia progression, while for lower-frequency bands there is a partial recovery of this variability going from PD-MCI to PDD.

## Discussion

It has been proposed that "dementia is a holistic clinical condition that does not emanate from a single region of a single FC [functional connectivity] network"^16^. In the case of our connectivity results, the holistic nature of dementia is apparent: mostly diffuse changes are noticeable in the correlation structure between disease classes. Such changes generally involved an increase in connectivity for the slower-wave bands and a non-linear trend for the higher frequencies. Inter-subject variability in connectivity showed, if anything, stronger trends than connectivity itself. This only underscores the need for methods capable of distilling key network properties out of this wide variability.

We found that the simultaneous MSTs (mostly dt = 0, but also dt = 1 for alpha) are dominated by a largely unchanging pattern closely similar to the pattern of parcel-adjacency for the AAL atlas. Likewise, the distribution of edge weights for MSTs at dt = 0 dramatically differs from that seen in the other time offsets, with many edges showing near-zero distance (much higher correlation). We suspect this homogeneity at dt = 0 is due to field-spread causing artifactual connectivity between adjacent parcels. We therefore subtracted out the difference in parcel correlations for the adjacent-parcel cases, but still found few interesting class-wise differences at dt = 0. At other time offsets, particularly in alpha with dt = 2, we found a prominent grouping of correlations centered in the parieto-occipital region. Strong alpha band activity in this region is typical during relaxed wakefulness, particularly with eyes closed^55^. Interestingly however, this grouping showed much weakened alpha correlations in the MCI group, and was nearly indistinguishable in the PDD cases (Fig. 6c). Outside of this, few distinctive parcel cross-peaks are seen across bands that would indicate a simple, conserved connectivity motif that distinguishes disease classes.

It has been reported that, compared with non-demented PD patients, people with PDD exhibit reduced connectivity specifically between the inferior frontal cortex and the cingulate (rows/columns 11–16 and 31–36, respectively, in the connectivity maps in Fig. 6)^16^. In addition, the orbito-frontal regions (channels 5–6, 9–10, 15–16) have an important role in PD progression with regard to network properties^25^. In contrast, our connectivity maps generally exhibit global changes in connectivity, with the sign of the change dependent upon the frequency band examined.

It is worth mentioning that the MST edge length distributions, in which we observed very high-significance differences between cognitive classes, are a kind of distillation of the correlation maps shown in Fig. 6. As the overall amount of correlation increases, the distances of the MSTs based on those correlations will tend to decrease. Therefore the KS-test results on the MST edge lengths (Supplementary Table S1) can be seen as giving a sense of the significance of the differences that are visible in these maps.

Using our correlation maps as a proxy for overall connectivity, we found strong increases in overall parcel correlation for the low-frequency bands, and large decreases for higher frequencies, with the alpha band as an intermediate case, showing a strong increase chiefly for the group of occipital-parietal parcels previously mentioned. It is tempting to connect the increase in low-frequency correlation with the widely observed increase in low-frequency power in PDD, though findings on this point have been inconsistent^9^, and in our case we are not measuring total power at all, but rather time-lagged correlation between parcels.

The class-average MSTs for higher time offsets, mainly for the beta band, showed a dramatic change in structure from a more locally connected random or "regular" network^1^ to one centered far more on a few very high degree nodes, much more consistent with a "scale-free" or "rich club" structure, in which a few nodes dominate the connectivity pattern. Changes in such "hubs" are thought to be highly important in cognitive dysfunction^1,6^. Yet, examination of the individual patient MSTs in these time offsets generally shows little consistency as to the location of these "hubs". In general, for dt > 0, the individual variations in MST structure become larger than the common features seen in the averaged MSTs. Furthermore, the failure to detect significant differences in degree distribution between classes (except in the theta-band) argues against any widespread change in scale-freeness.

The beta band is an interesting exception to this general lack of pattern; here, we instead observe a pattern reminiscent of the "hub overload" model^1^: although the overall node-degree distribution is not significantly changed, the PD-NC group shows a single strongly connected hub in the occipital lobe, the PD-MCI group shows a (perhaps compensatory) partial shift to alternate hubs mainly in the postcentral gyrus and the frontal lobe, and the PDD group shows a final weakening or disappearance of these hubs (Fig. 1). Such shifting of beta band information flow from the occipital to frontal/temporal regions was previously reported^20^; it is worth noting that other work, however, has found changes in information integration in Parkinsonian dementia solely in the alpha1 band^56^.

Considering the shortest-path length distributions for the three cognitive classes, the beta band again shows a particularly stepwise progression with increasing dementia severity (Fig. 2d), with both increasing mean path-length and greater variance, suggesting both a decrease in connection efficiency and increasing disorganization or diversity of pathways. The trend in the other bands is more puzzling, however, and may indicate a previously unrecognized role in dementia progression. Theta and gamma appear to show major changes for the MCI group alone, which may indicate that a particular network scale becomes predominant or catalytic during this intermediate phase of dementia. The distribution of clustering coefficients, which tracks roughly inversely to the path lengths distribution, may be rationalized as follows: both shorter path lengths and higher clustering imply a more efficient information-transferring structure.

To our knowledge, little or no attention has been given to the level of inter-patient variation in different grades of cognitive decline. Yet the differences in variance between cognitive groups (Fig. 7) were marked, suggesting a global convergence of correlation structure in most bands (i.e., decreasing variability with dementia severity) contrasted with a divergence in the low-frequency bands. While this phenomenon does not lend itself to individual-level diagnostics based on connectivity (indeed, it suggests a limitation to them), it may hold promise for better understanding of the etiology and mechanism of PD-related cognitive decline.

Our use of MVR and MST class-adjacency statistics, and a permutation-based method to generalize MVR to more than two classes, showed extremely significant pairwise class differences through MVR, while also observing very marked differences in class-adjacency between the three cognitive classes. In particular, the fact that the PD-NC and PD-MCI groups shared the largest number of hybrid edges overall, and PD-NC and PDD the lowest (Fig. 5) is consistent with what might be expected of a progressive disease, where PDD represents much more serious impairment than MCI, although such conclusions are limited by the cross-sectional nature of our data. Though these results are tentative, the relative “closeness” of the PD-NC and PD-MCI nodes in the MST suggests that the most marked changes in brain connectivity (as distinct from activation) instead occur during the progression from PD-MCI to PDD, rather from PD-NC to PD-MCI. To our knowledge, this is the first use of MST class-adjacencies as a tool for analyzing relative disease group differences.

In general, class-adjacency may help expand upon the limited information provided by the MVR, by estimating graphically not just that *some* classes are drawn from different distributions, but also which classes are more or less different. For the three-way MVR cut, 5 of the 15 possible combinations of band and dt (essentially, all of the dt = 0 cases) showed only very small (< 2 SD) from the expected number of total trees; however, these all still showed highly significant deviations from expectation in the individual elements of the class adjacency matrix (Supplementary Table S2).

Though the relative sizes of the deviations in the class-adjacency elements closely paralleled the corresponding pairwise MVRs, deviations were more poorly resolved in the latter, most likely because pairwise tests cannot take into account simultaneous, hierarchical associations and contexts that exist between multiple classes; the multi-class approach, therefore, may be inherently more powerful at establishing class differences. We believe this underscores the potential utility of the class-adjacency approach to multi-class MST analysis for detecting structure and clustering between data classes.

Overall, our approach is novel in being one of the few examples of graph-theoretical connectivity analyses applied to MEG data, and the first attempt at an approximate generalization of the MVR concept to > 2 classes. It is also, to our knowledge, the only MEG study that applies the MVR or class-adjacency approach to detect differences in connectivity between brain disease states. Our finding that the MST-based approaches, particularly 3-class MVR, were relatively robust to confounders such as patient age and levodopa intake also suggests that combining MSTs with edge-cut testing may be a substantially more robust way to analyze functional connectivity than by correlations alone.

We have pursued MSTs because they avoid the inherent arbitrariness of threshold choice in other forms of connectivity analysis, which has often led to conflicting findings in the literature, particularly regarding network features^1^. By providing a simple and objective standard for connection selection, the MST offers some hope of decisively resolving this bias and arbitrariness^1,7,10,11^. Similarly, the multivariate-runs approach offers promise because of its simplicity and its avoidance of arbitrary thresholds.

Differences between imaging technologies can exacerbate differences in local connectivity in many graph methods, so that even studies using the same imaging method sometimes show contradictory network effects^1^. We suggest that the use of k-MSTs and MVR can considerably alleviate this limitation, by drawing on a basket of shortest-paths instead of the single absolute shortest path.

There have been concerns about the "curse of dimensionality" in the application of the MVR test, especially in cases where the underlying distributions differ mainly in their variance ("scale alternatives")^57^. It is proposed in^57^ that test power can be improved by including the second- through fifth-order MST connections (an nth-order MST is derived by removing the connections in the first n-order MSTs and calculating a new MST from the connections that remain). In this case, we found the test performed adequately using just the first-order MST alone, however this may be a useful future direction.

In conclusion, we have used MST-based metrics, including the multivariate-runs test and MST class-adjacency statistics, to detect clear differences in network properties and underlying distributions associated with different stages of Parkinsonian cognitive decline. Future work may focus on ways to validate and combine these tests in order to develop more accurate, non-invasive models of PD and other brain-connectivity diseases.

## Supplementary Information

Supplementary Information.

## Abbreviations

PD-NC: Parkinson’s disease, normal cognition
PD-MCI: Parkinson’s disease with mild cognitive impairment
PDD: Parkinson’s disease with dementia
MST: Minimum spanning trees
MVR: Multivariate runs
