# Supplementary material for: A novel approach to understanding Parkinsonian cognitive decline using minimum spanning trees, edge cutting, and magnetoencephalography
Source: Sci Rep. 2021 Oct 5;11:19704. doi: 10.1038/s41598-021-99167-2 (PMC8492620; doi:10.1038/s41598-021-99167-2)
Supplement: Supplementary file 1 — Supplementary Information. [file 41598_2021_99167_MOESM1_ESM.pdf]

Supplementary information for "**A Novel Approach to Understanding Parkinsonian Cognitive Decline Using Minimum Spanning Trees, Edge Cutting, and Magnetoencephalograpy** "

Olivier B. Simon, PhD, Isabelle Buard, PhD, Donald C. Rojas, PhD, Samantha K . Holden, MD MS, Benzi M. Kluger, MD MS, Debashis Ghosh, PhD

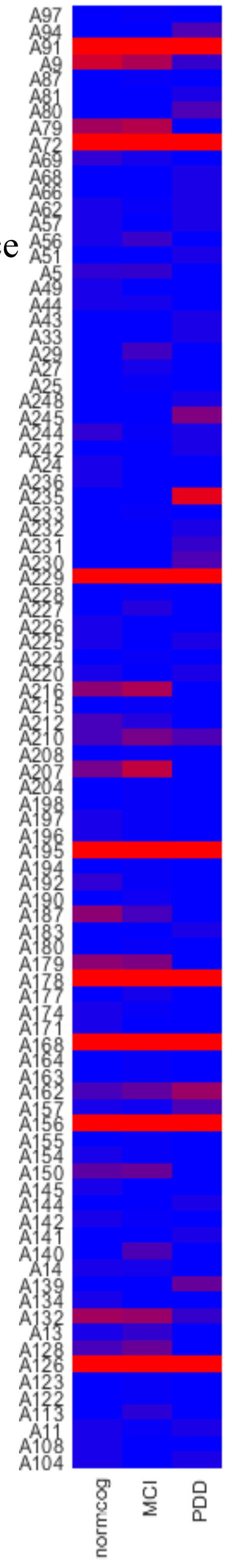

Supplementary Figure S1. Heatmap of those MEG channels that were dropped from the analysis at least once displayed with respect to cognitive group. Blue indicates the least number of times the channel was dropped, while red indicates channels that were dropped for all patients. In general the number and pattern of dropped channels is similar across all three groups.

Supplementary Figure S2. 3-class MST edge-cut/MVR method is resistant to the confounding effects of patient age. Panels **a** and **b** show inter-parcel correlation variance maps for patients with above-median and below-median age, respectively. Here, the trend of decreasing average parcel correlation variance with cognitive decline is found almost exclusively in the patients with above-median age. In contrast, 3-sample MVR on the pooled MST for above- and below-median age (panels **c** and **d** respectively) reveals that parcels from the same cognitive group continue to group together exclusively within the MST regardless of age, with almost no "hybrid" edges between dissimilar classes. Results are shown for theta band with dt=2; similar effects are observed for the other brainwave frequency bands.

a)

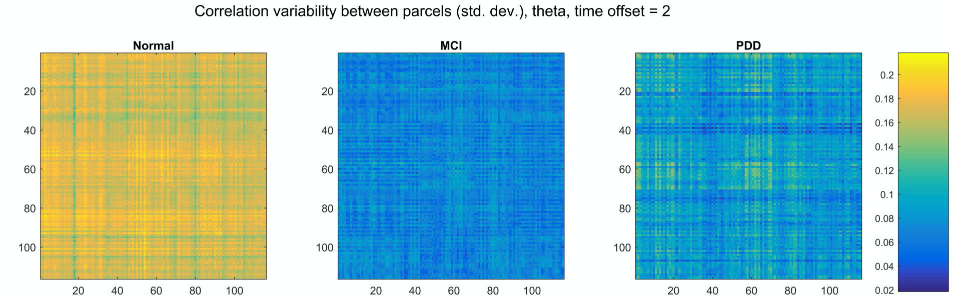

b)

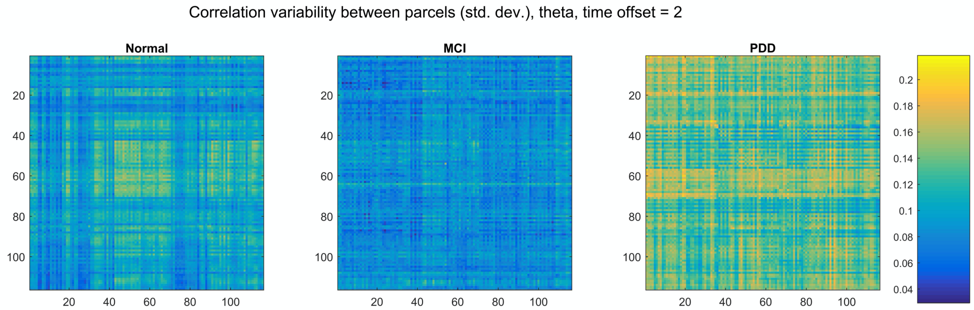

c)

d)

Class average correlation-distance MST, 3-way cut, theta, time offset = 2:
(Normal, red; MCI, green; PDD, blue)

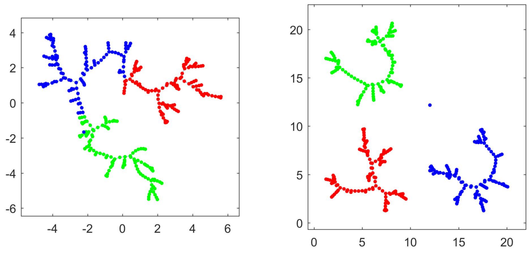

Class average correlation-distance MST, 3-way cut, theta, time offset = 2:
(Normal, red; MCI, green; PDD, blue)

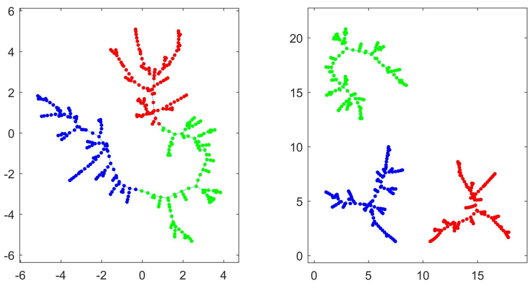

Supplementary Figure S3. 3-class MST edge-cut/MVR method is resistant to the confounding effects of patient L-dopa equivalent dosage. Panels **a** and **b** show inter-parcel correlation variance maps for patients with above-median and below-median dosage, respectively. Here, the trend of decreasing average parcel correlation variance with cognitive decline is found almost exclusively in the patients with above-median dosage. In contrast, 3-sample MVR on the pooled MST for above- and below-median dosage (panels **c** and **d** respectively) reveals that parcels from the same cognitive group continue to group together exclusively within the MST regardless of dosage, with almost no "hybrid" edges between dissimilar classes. Results are shown for beta band with dt=1; similar effects are observed for the other brainwave frequency bands.

a)

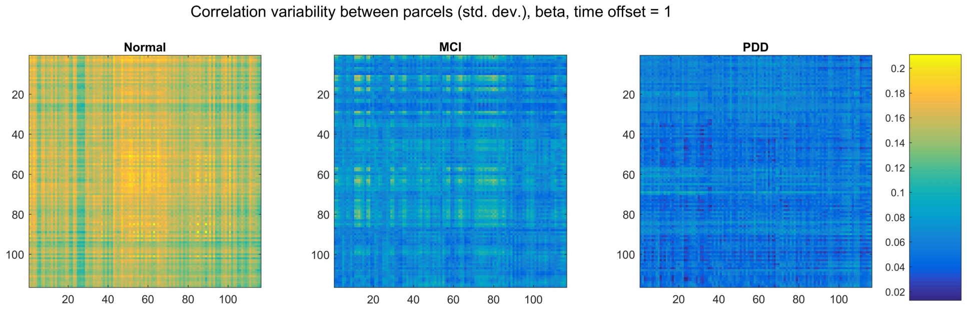

b)

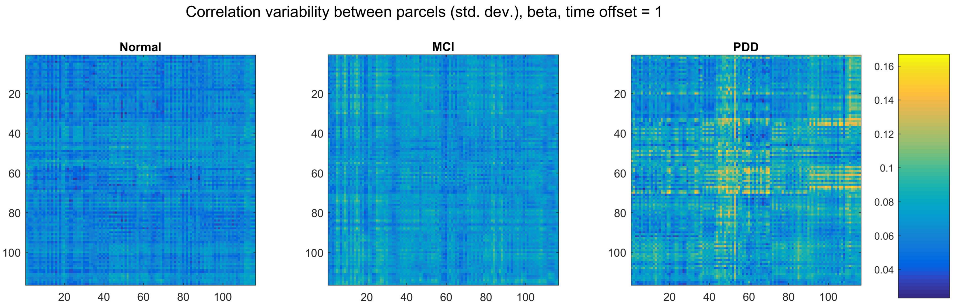

c)

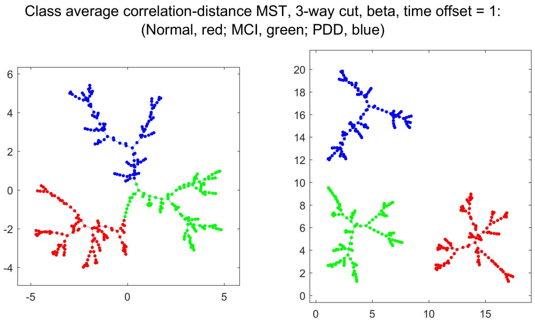

d)

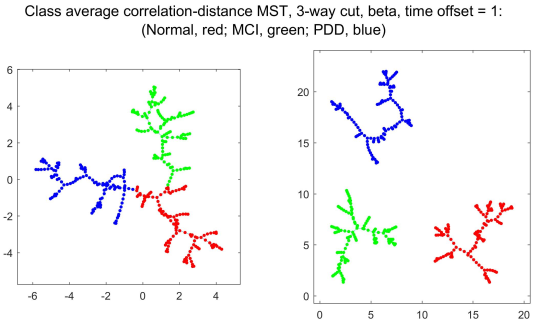

Supplementary Figure S4. Further visual examples of the 3-way class adjacency procedure, for beta band and time offset dt=1. Figure **a** compares normal controls and MCI cases. Figure **b** compares MCI and PDD cases. Figure **c** compares PDD cases to normal controls. In each subfigure, the left panel shows the MST constructed over the pooled inter-parcel correlations, while the right panel shows the fragments resulting from the cutting of edges between dissimilar nodetypes.

a)

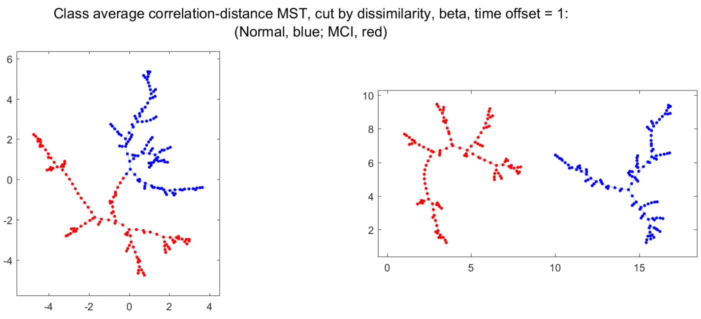

b)

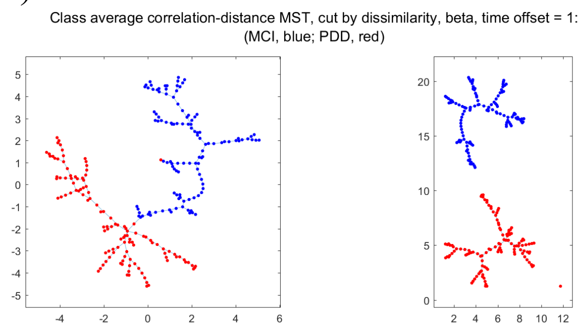

c)

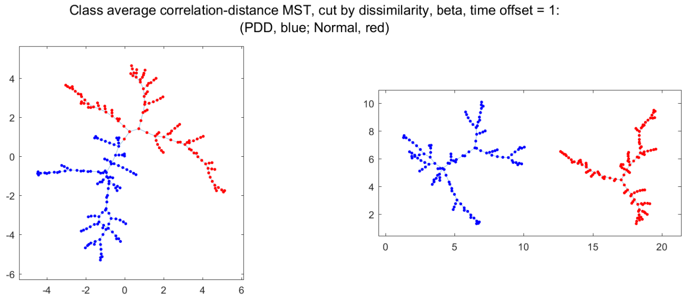

d)

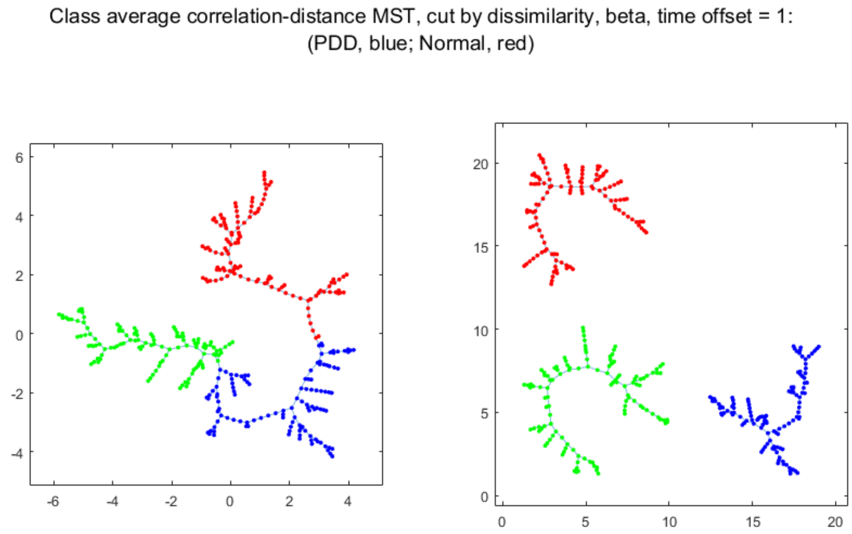

Supplementary Table S1. Selected KS test p-values for a) edge-lengths, b) node degree distribution (Bonferroni-corrected for number of band/interval/class pair combinations), c) shortest path lengths, and d) clustering coefficients, for combinations of frequency, dt, and class pairing that reached highest levels of significance. "N"= normal cognition, "M"= MCI group, "P"= PDD group.

a)

Band/interval/classes	Edge Lengths
**Theta, dt=1, M-P**	3.30E-53
**Theta, dt=1, N-P**	2.58E-97
**Theta, dt=2, M-P**	8.30E-54
**Theta, dt=2, N-P**	2.85E-66
**Gamma, dt=1, N-M**	1.12E-94
**Gamma, dt=2, M-P**	1.07E-77

b)

Band/interval/classes	Node Degrees
**Theta, dt=1, N-M**	1.86E-05
**Theta, dt=1, N-P**	1.37E-04

c)

Band/interval/classes	Shortest Path Lengths
**Delta, dt=0, M-P**	2.11E-161
**Theta, dt=0, N-P**	1.36E-114
**Theta, dt=2, N-M**	<1e-197
**Theta, dt=2, M-P**	<1e-197
**Theta, dt=2, N-P**	5.47E-132
**Alpha, dt=1, M-P**	2.66E-164
**Alpha, dt=1, N-P**	1.12E-72
**Alpha, dt=2, N-M**	3.48E-70
**Alpha, dt=2, M-P**	<1e-197
**Alpha, dt=2, N-P**	<1e-197
**Beta, dt=1, N-M**	<1e-197
**Beta, dt=1, M-P**	1.3496e-197
**Beta, dt=1, N-P**	<1e-197
**Beta, dt=2, N-M**	<1e-197
**Beta, dt=2, M-P**	<1e-197
**Gamma, dt=1, N-M**	<1e-197
**Gamma, dt=1, M-P**	1.7346e-253
**Gamma, dt=1, N-P**	<1e-197
**Gamma, dt=2, N-M**	<1e-197
**Gamma, dt=2, M-P**	<1e-197
**Gamma, dt=2, N-P**	4.9178e-125

d)

Band/interval/classes	Clustering Coefficients
**Delta, dt=2, N-P**	5.04E-06
**Theta, dt=2, M-P**	3.76E-04
**Alpha, dt=2, M-P**	9.8412e-06
**Alpha, dt=2, N-P**	6.5598e-11
**Beta, dt=1, N-M**	6.48E-04
**Beta, dt=1, N-P**	5.4468e-09
**Gamma, dt=1, N-P**	0.003
**Gamma, dt=2, N-M**	1.89E-05

Supplementary Table S2. 2- and 3-way MVR results, showing standard deviations from expectation for each frequency/time offset pairing for total trees. In general, large negative deviations in the 2-way MVR cuts are matched by large negative values in the 3-way cut, and inversely.

	2-class			3-class
	N-M	M-P	N-P	
**delta, dt=0**	1.71	-1.32	0.53	1.26
**delta, dt=1**	-4.87	-11.59	-11.99	-14.19
**delta, dt=2**	-15.02	-15.02	-15.02	-26.17
**theta, dt=0**	-4.08	-1.84	0.40	-0.68
**theta, dt=1**	-11.07	-14.89	-14.10	-21.85
**theta, dt=2**	-15.02	-15.02	-15.02	-26.08
**alpha, dt=0**	-0.13	0.92	-2.50	0.23
**alpha, dt=1**	-8.04	-11.20	-14.36	-16.99
**alpha, dt=2**	-15.02	-15.02	-15.02	-26.20
**beta, dt=0**	0.92	0.00	-0.79	0.69
**beta, dt=1**	-15.02	-14.62	-14.89	-25.95
**beta, dt=2**	-15.02	-14.89	-14.89	-25.74
**gamma, dt=0**	1.05	-0.66	-0.79	1.04
**gamma, dt=1**	-15.02	-15.02	-15.02	-26.22
**gamma, dt=2**	-15.02	-14.62	-15.02	-25.85

Supplementary Table S3. 3-way class-adjacency results from cutting the pooled MST of all three dementia classes (N = normal cognition, M = mild cognitive impairment, P = Parkinsonian dementia). For each pairing of brainwave band and time-offset (dt), the number of edges resulting from each possible pairing of classes is presented, with the corresponding standard deviations from expectation presented below. (The upper right three entries are blanked out for clarity, since the class-adjacency matrix is symmetrical.)

delta, dt=0	N	M	P	
N	36	~	~	
M	116	13	~	
P	90	37	55	
# subtrees	80	103	61	244

	N	M	P
N	-0.4776		
M	5.4379	-5.1843	
P	1.797	-5.7497	3.403

theta, dt=0	N	M	P	
N	88	~	~	
M	38	14	~	
P	109	79	19	
# subtrees	28	102	97	227

	N	M	P
N	9.883		
M	-5.5147	-4.8373	
P	4.4445	0.2432	-3.8811

alpha, dt=0	N	M	P	
N	90			
M	90	13		
P	38	106	10	
# subtrees	26	103	106	235

	N	M	P
N	10.6347		
M	1.7935	-5.2413	
P	-5.6398	4.0958	-5.8436

delta, dt=1	N	M	P	
N	75	~	~	
M	77	69	~	
P	14	17	95	
# subtrees	41	47	21	109

	N	M	P
N	7.6884		
M	-0.0426	6.426	
P	-9.0889	-8.64	11.9187

theta, dt=1	N	M	P	
N	108	~	~	
M	32	91	~	
P	8	1	107	
# subtrees	8	25	9	42

	N	M	P
N	14.4345		
M	-6.4846	10.9761	
P	-9.918	-10.9367	14.2543

alpha, dt=1	N	M	P	
N	92			
M	53	72		
P	3	27	100	
# subtrees	24	44	16	84

	N	M	P
N	11.3484		
M	-3.5135	7.1044	
P	-10.6725	-7.2313	13.0724

delta, dt=2	N	M	P	
N	114	~	~	
M	2	115	~	
P	0	1	115	
# subtrees	2	1	1	4

	N	M	P
N	15.916		
M	-10.8444	16.1126	
P	-11.0788	-10.949	16.0015

theta, dt=2	N	M	P	
N	115	~	~	
M	1	115	~	
P	2	0	114	
# subtrees	1	1	2	4

	N	M	P
N	15.3328		
M	-10.7894	15.4025	
P	-10.5323	-10.8723	15.1522

alpha, dt=2	N	M	P	
N	115			
M	1	115		
P	0	2	114	
# subtrees	1	1	2	4

	N	M	P
N	15.3967		
M	-10.7188	15.4217	
P	-10.8526	-10.6116	15.1835

beta, dt=0	N	M	P	
N	75	~	~	
M	109	17	~	
P	40	90	16	
# subtrees	41	99	100	240

	N	M	P
N	7.4902		
M	4.499	-4.3486	
P	-5.2528	1.7823	-4.5581

gamma, dt=0	N	M	P	
N	82	~	~	
M	112	6	~	
P	43	86	18	
# subtrees	34	110	98	242

	N	M	P
N	8.849		
M	4.8829	-6.4829	
P	-4.816	1.2262	-4.108

beta, dt=1	N	M	P	
N	115	~	~	
M	1	115	~	
P	2	3	111	
# subtrees	1	1	5	7

	N	M	P
N	15.5905		
M	-10.8158	15.6317	
P	-10.5849	-10.5242	14.7972

gamma, dt=1	N	M	P	
N	115	~	~	
M	1	115	~	
P	1	0	115	
# subtrees	1	1	1	3

	N	M	P
N	15.5158		
M	-10.7714	15.5899	
P	-10.8059	-10.9994	15.6335

beta, dt=2	N	M	P	
N	115	~	~	
M	1	115	~	
P	2	3	111	
# subtrees	1	1	5	7

	N	M	P
N	15.5009		
M	-10.7309	15.4785	
P	-10.554	-10.4724	14.6206

gamma, dt=2	N	M	P	
N	115	~	~	
M	1	115	~	
P	0	5	111	
# subtrees	1	1	5	7

	N	M	P
N	15.5359		
M	-10.712	15.4888	
P	-10.9165	-10.2135	14.7515
